# Personalized repetitive transcranial magnetic stimulation temporarily alters default mode network in healthy subjects

**DOI:** 10.1038/s41598-019-42067-3

**Published:** 2019-04-04

**Authors:** Aditya Singh, Tracy Erwin-Grabner, Grant Sutcliffe, Andrea Antal, Walter Paulus, Roberto Goya-Maldonado

**Affiliations:** 1 Systems Neuroscience and Imaging in Psychiatry, Department of Psychiatry and Psychotherapy of the University Medical Center Göttingen, Göttingen, Germany; 2 Department of Clinical Neurophysiology of the University Medical Center Göttingen, Göttingen, Germany; 3 Institute of Medical Psychology, Otto-von-Guericke University Magdeburg, Magdeburg, Germany

## Abstract

High frequency repetitive transcranial magnetic stimulation (HF-rTMS) delivered to the left dorsolateral prefrontal cortex (DLPFC) is an effective treatment option for treatment resistant depression. However, the underlying mechanisms of a full session of HF-rTMS in healthy volunteers have not yet been described. Here we investigated, with a personalized selection of DLPFC stimulation sites, the effects driven by HF-rTMS in healthy volunteers (n = 23) over the default mode network (DMN) in multiple time windows. After a complete 10 Hz rTMS (3000 pulses) session, we observe a decrease of functional connectivity between the DMN and the subgenual Anterior Cingulate Cortex (sgACC), as well as the ventral striatum (vStr). A negative correlation between the magnitude of this decrease in the right sgACC and the harm avoidance domain measure from the Temperament and Character Inventory was observed. Moreover, we identify that coupling strength of right vStr with the DMN post-stimulation was proportional to a decrease in self-reports of negative mood from the Positive and Negative Affect Schedule. This shows HF-rTMS attenuates perception of negative mood in healthy recipients in agreement with the expected effects in patients. Our study, by using a personalized selection of DLPFC stimulation sites, contributes understanding the effects of a full session of rTMS approved for clinical use in depression over related brain regions in healthy volunteers.

## Introduction

Despite its increasing relevance for clinical use, the basic underlying mechanism of action of repetitive transcranial magnetic stimulation (rTMS) protocols remains unexplored in healthy subjects for a complete session of 10 Hz rTMS (3000 pulses in 37.5 min), which is the first FDA approved treatment protocol for treatment resistant depression. Much variability in the resulting rTMS response exists, which could be reduced by accurate brain targeting based upon individual subject’s resting state functional magnetic resonance imaging (rsfMRI). Independent from the confounding effects of depression related symptomatology, an increased comprehension of the underlying neural mechanisms of a full session of HF-rTMS in a healthy human brain will help to better inform future research into the depressed brain and its treatment. While several studies have investigated the effects of rTMS in healthy volunteers^1–4^, the mechanism of action of the complete session of 10 Hz rTMS with up to 1 hour observation of neural effects has not yet been performed.

The default mode network (DMN) is a network consisting of the medial and lateral temporal lobe, medial prefrontal cortex and medial and lateral parietal cortex. It has been implicated in the pathophysiology of depression^5–17^ and its hyperconnectivity to the subgenual Anterior Cingulate Cortex (sgACC) has been reported to be normalized after successful rTMS treatment^5,18^. A recent study^19^ explored in healthy subjects the mechanism of about a third of the 10 Hz rTMS protocol (1200 pulses in 10 min) typically used to treat depression. The authors reported an increase in the functional connectivity of the sgACC to another network, consisting of the dorsal cingulate cortex, posterior dorsomedial prefrontal cortex, dorsolateral prefrontal cortex (DLPFC), inferior parietal lobule, inferior frontal cortex and posterior temporal lobes, but no changes to a network resembling the DMN. Here, we aimed to address the mechanism of action of a complete session of 10 Hz rTMS, targeting individually selected left DLPFC stimulation sites and precisely assisted by online neuronavigation.

Fitzgerald and colleagues^20^ investigated different methods of targeting and stimulating the DLPFC. They concluded that targets defined on the 10–20 electroencephalographic (EEG) system or those selected with the use of individual structural MRI images and a neuronavigation system are spatially more precise than those selected with the “standard procedure”, referring to the scalp location 5 cm anterior to the motor cortex^21^. Additionally reinforcing the use of neuroimaging techniques to select stimulation sites, studies revealed that the antidepressant response of left DLPFC stimulation can be predicted by the negative functional connectivity with the sgACC^22,23^. Therefore, to personalize HF-rTMS stimulation sites in healthy volunteers, we integrated the individual variation captured by rsfMRI^24^ in a protocol that combined spatial and temporal information from independent components that best represented the left DLPFC and the sgACC regions.

In this study we evaluated the DMN by using multiple rsfMRI windows before and after stimulation in a double blind (interviewer and participant), crossover, and sham controlled study with healthy subjects. We argue that insights from one session of 10 Hz rTMS stimulation in healthy subjects can be relevant for a better understanding of the HF-rTMS antidepressant treatment, further prompting development of more efficient rTMS interventions. Such an application of single dosage of HF-rTMS in healthy subjects is unprecedented and the ~1 hour follow up using rsfMRI post stimulation will expectedly reveal pertinent insights in to the action of HF-rTMS. Reasoning that healthy subjects have normal baseline connectivity, we hypothesized that a full HF-rTMS session would change the functional connectivity of the sgACC to the DMN. If the underlying mechanism of 10 Hz rTMS at left DLPFC in healthy volunteers is analogous to that reported in patient population, then we would expect to see a decrease in functional connectivity of the sgACC to DMN. The harm avoidance (HA) dimension of the Temperament and Character Inventory (TCI) has been related to the activity of sgACC in adolescents^25^, healthy adults^26,27^ and to pharmacological antidepressant responses in adults^28–30^. Therefore, we selected this dimension of personality, as well as the Positive and Negative Affect Schedule (PANAS) to investigate how HF-rTMS driven effects would relate to phenotypic information from the healthy participants. We hypothesized that HA scores would anticipate DMN changes reaching the sgACC. Lastly, in agreement with previous studies that have shown that activity in sgACC is associated with sad mood^25,31,32^ and rumination^13^ in healthy subjects, we speculated that rTMS induced decoupling of sgACC and DMN would result in a decrease in the self-rated negative affect of participants after stimulation.

## Results

Only 1 subject was excluded due to not tolerating the stimulation, therefore data from 23 subjects were included in the final analysis. The mean age of the subjects was 25.8 ± 5.5 years with 9 female subjects.

### Target reproducibility

**Figure 1:**
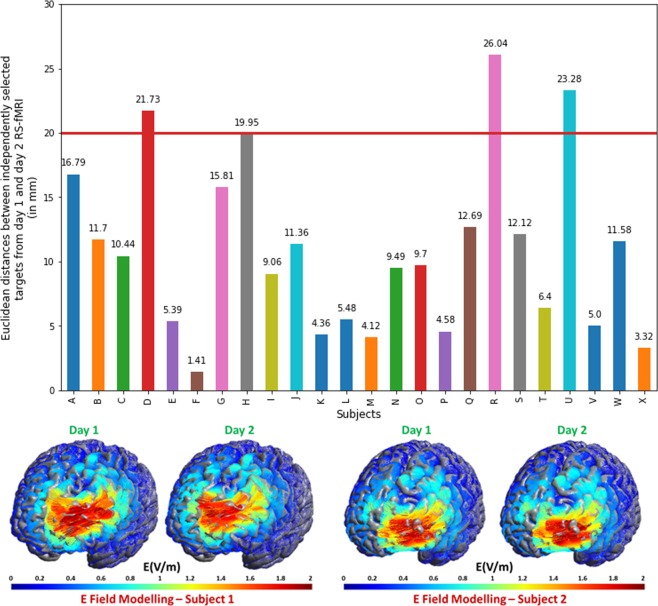
Distances between targets from day 1 and day 2 rsfMRI (n = 24) and electric field modelling for two example subjects. Upper panel: Bar plot of Euclidean distance between targets from day 1 rsfMRI and targets from day 2 rsfMRI. 3 subjects have a distance larger than 20 mm. Lower panel: A qualitative description of electric field modelled (Thielscher *et al*., 2015) on the two targets from day 1 and day 2 rsfMRI data. A quick look reveals the similarity of the electric field when either of the targets are used.

To establish the consistency of the rsfMRI based method for personalized target selection, one must first test whether target sites generated from different datasets of the same subject will concur. To test the reproducibility of our target selection process, we repeated the day 1 steps of target selection also on pre-stimulation rsfMRI data from the day on which real stimulation was delivered. We then calculated the Euclidean distances between targets selected from baseline and stimulation day. The mean distance between the two was 10.9 mm (6.74 mm SD), which was significantly less than 20 mm (two tailed, one sample t test, t value = −6.61, p < 0.0001) as presupposed in the literature (see Discussion). Figure 1, upper panel presents a bar plot of the distances between the targets for each subject. There are only three subjects for whom the distances between the day 1 and day 2 targets are slightly above 20 mm (diameter of the electric field sphere covered by figure-of-eight coils)^33^. Figure 1, lower panel, presents a qualitative comparison of the modelled electric field (SimNIBS^34^) generated by stimulation at targets for two example subjects.

### Target quality

**Figure 2:**
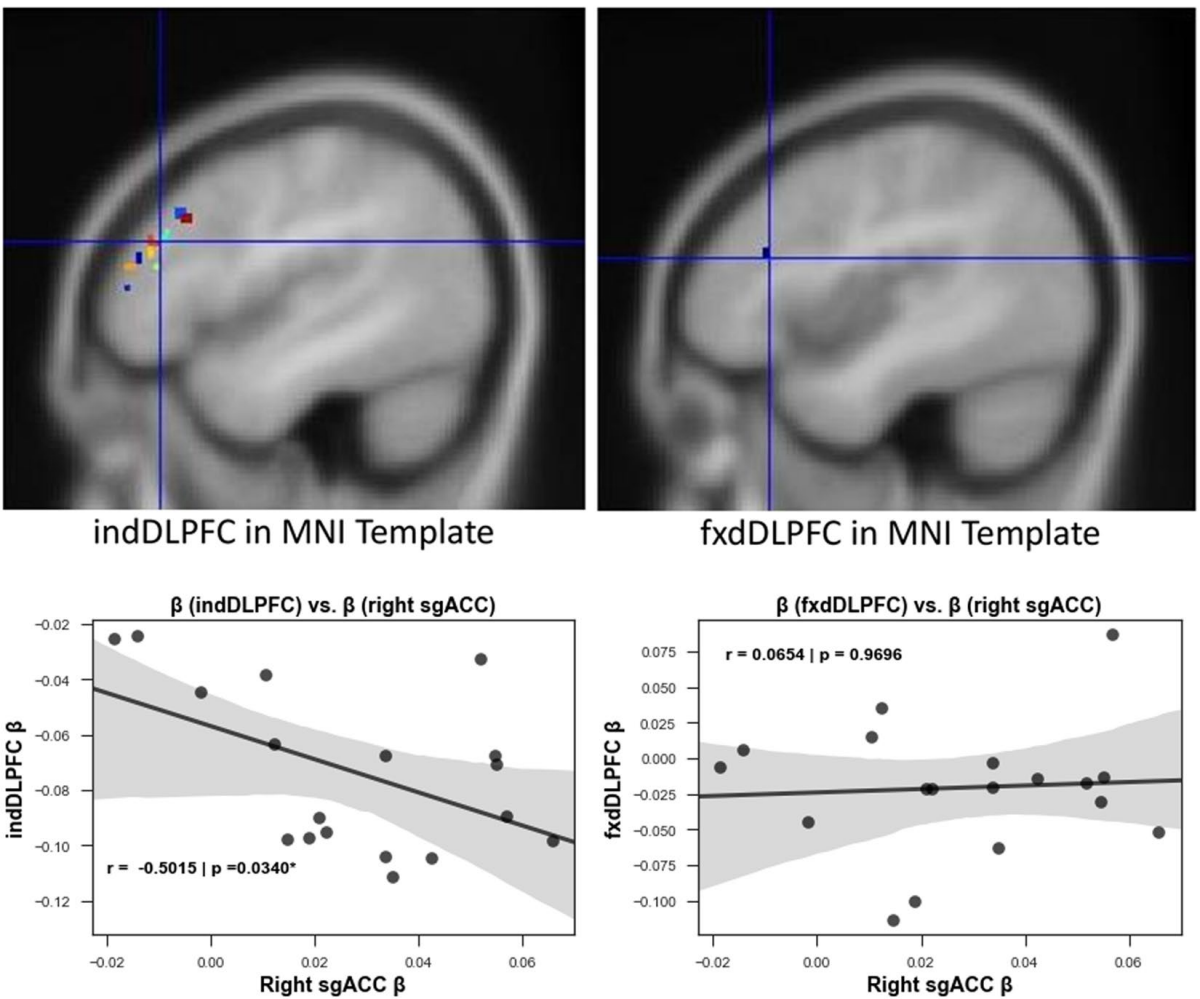
Left DLPFC targets and their correlation with right sgACC. Upper panel: Figure showing the 2 mm radius ROIs for targets obtained by the selection process described in the study (n = 22) [left] and the ROI of 2 mm radius around the standard MNI DLPFC coordinates [right]. Lower panel: The plot on the left shows that targets identified using the described selection process have a much higher negative correlation with right sgACC (r = −0.5015, *p < 0.05) than when the standard MNI left DLPFC coordinates are used (right plot). Since, antidepressant response of rTMS is linked to the connectivity of stimulated site and the sgACC; individualized targets would be promising for better therapeutic response than standard targets (n = 18).

The other important aspect of our target selection process is the quality of the target that it yields. Effective HF-rTMS stimulation for the treatment of depression using left DPLFC targets is achieved by stimulating at targets with higher negative functional connectivity to sgACC^22,23^. Thus, we calculated the functional connectivity between the targets selected by our method (named individual DLPFC, indDLPFC) and the right sgACC, and also between fixed MNI coordinates based left DLPFC (named fxdDLPFC) and the right sgACC. We used 2 mm radius ROI spheres to extract the betas from DLPFC regions. In Fig. 2, upper panel, note that indDLPFC targets spread across the left DLPFC and having a larger radius would have caused overlaps with the fxdDLPFC target, potentially diluting specific correlations with right sgACC. We preserved with 5 mm radius of the latter as in Tik *et al*.^19^ for comparison. We found a higher negative connectivity between indDLPFC and right sgACC compared to that between fxdDLPFC and right sgACC (Fig. 2, lower panel), thus implying that indDLPFC can be more promising therapeutically as compared to fxdDLPFC.

### Behavioural scales results

**Figure 3:**
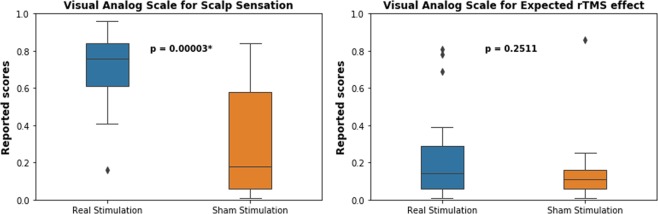
Physical sensation and resultant expectation about the efficacy of rTMS stimulation in subject (n = 17). The plot on the left shows that the scalp discomfort experienced by the subjects was significantly higher during the real than sham condition. However, their internal expectation of the induced changes in their mental states did not differ across real and sham stimulation sessions.

As would be expected, we did not see any differences in healthy volunteers in the MADRS, HAM-D, YMRS, PANSS, and BDI II scores between day 2 and day 3 of stimulation. However, subjects had different physical sensations arising from the two sessions (two sample t test, t = 4.89, p < 0.0001), although they expected both stimulation sessions to be equally effective (two sample t test, t = 1.17, p > 0.05, see Fig. 3), as measured by visual analogue scales. This implies that our method of blinding successfully maintained similar levels of internal expectation between real and sham rTMS even though they experienced a difference in scalp discomfort. We believe the successful blinding was a result of nullification of external expectations of subjects. Since the subjects would have intuitively expected higher scalp sensation to lead to higher rTMS effects, we instructed the subjects that our study was testing out two different stimulation coils.

### RsfMRI functional connectivity changes post stimulation

**Figure 4:**
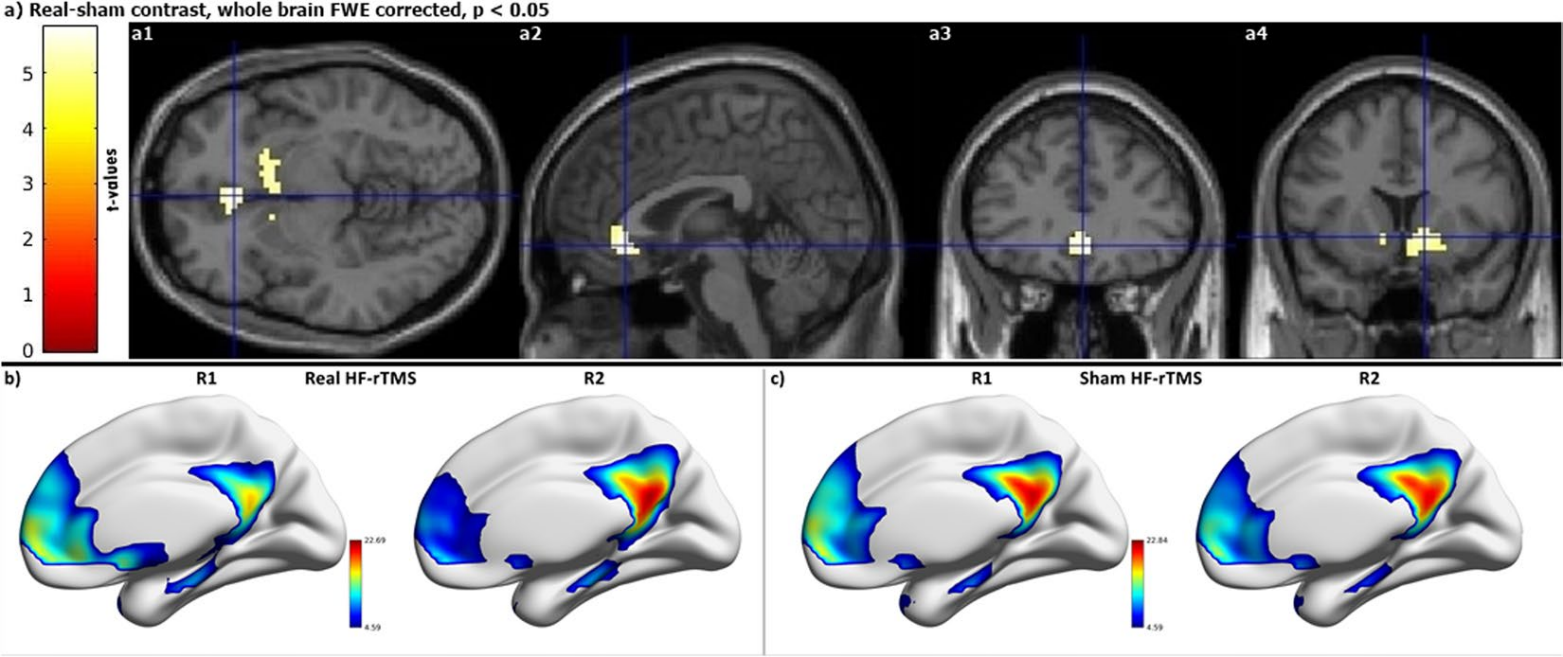
Functional connectivity decrease is only seen after real HF-rTMS (n = 23). (**a**) Decreased functional connectivity in bilateral sgACC (a1 to a3) and vStr (a4) during R2 when compared to R1. (**b**) Default mode network after real HF-rTMS during R1 and R2. (**c**) Default mode network after sham HF-rTMS during R1 and R2. Colorbars represent t-values.

To exclude the possibility of false positive functional connectivity results arising from nuisance movement, we compared the root mean square of frame wise head displacement parameters^35^ from individual frames. We found that the extent of motion did not differ across rsfMRI time windows for real and sham conditions. To investigate the mechanisms of HF-rTMS over the DMN, we contrasted this network during the four rsfMRI scanning sessions in real versus sham conditions. We observed a robust decrease in functional connectivity specifically involving the sgACC (Fig. 4 a1–a3) and the ventral striatum (vStr) (Fig. 4 a4) bilaterally during the R2-R1 contrast. This decrease in functional connectivity weakened over time and the connectivity in the ventral striatum returned to baseline level already in R3, while the decrease in the sgACC coupling persisted until R3, albeit less pronounced than during R2. Also, important to note is that this decrease in functional connectivity is only seen in the real HF-rTMS stimulation condition, but not during the sham HF-rTMS.

**Figure 5:**
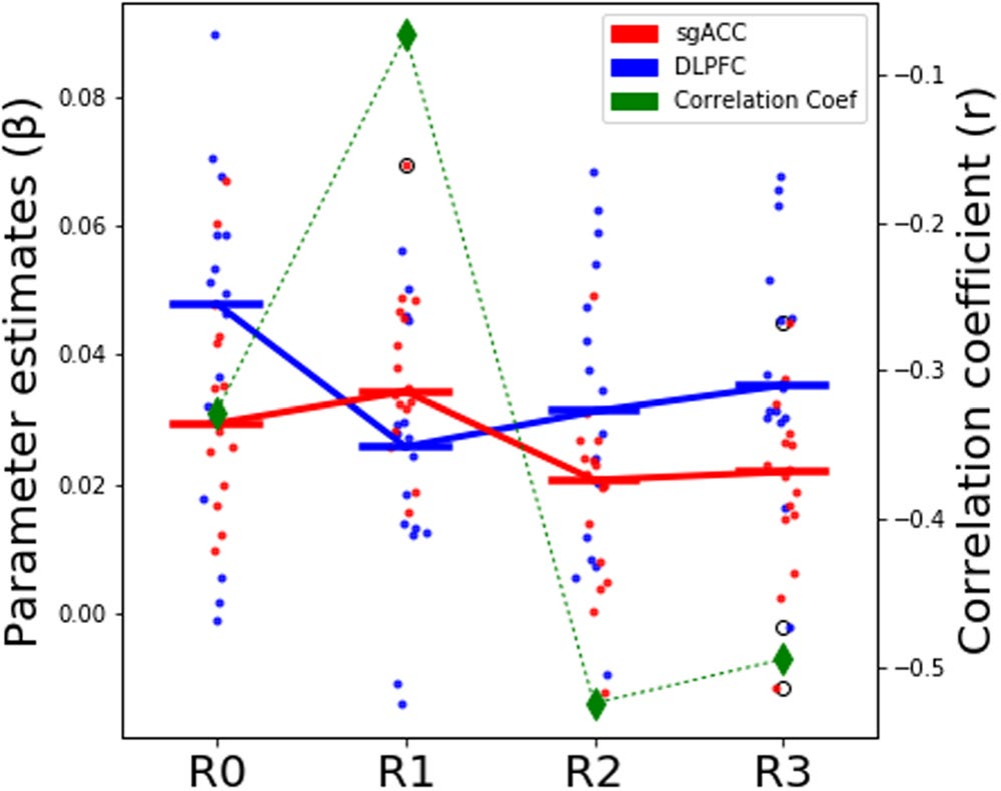
Plotting the interaction of personalized left DLPFC with sgACC and DMN. The plot shows the parameter estimates of left DLPFC functional connectivity to IC-DLPFC network and that of sgACC to DMN. The bars represent the median value of the parameter estimates. The plot shows how left DLPFC changes in functional connectivity occur during R1 rsfMRI session. While the sgACC functional connectivity changes follow later during R2 rsfMRI session. The green dashed line plots the correlation coefficients (right y-axis) between the parameter estimates of left DLPFC and sgACC. It shows that the baseline negative correlation between left DLPFC and sgACC is reduced after stimulation in R1 rsfMRI session, followed by stronger negative correlation between the two regions during R2 and R3 rsfMRI sessions. Refer to legend for info on color coding.

We also explored the functional connectivity of the personalized left DLPFC stimulation site with that of sgACC as well as the DMN. The parameter estimates of the connectivity strength of personalized left DLPFC to the IC-DLPFC network and of sgACC to DMN is plotted in Fig. 5 (left y-axis). It shows that the functional connectivity of left DLPFC decreases from R0 to R1. This is followed by a decrease in the functional connectivity of the sgACC from R1 to R2. The difference in time windows during which the functional connectivities of left DLPFC and sgACC decrease, shows how the HF-rTMS effect spreads from left DLPFC to sgACC across time.

In addition, the correlation between the left DLPFC and sgACC was evaluated. The green dashed line plots the correlation coefficients (right y-axis) between the parameter estimates of left DLPFC and the sgACC during the four rsfMRI sessions (R0 to R3). It shows that the baseline negative correlation between left DLPFC and sgACC reduces during R1 but returns to more negative values during R2 and R3.

### A predictor for rTMS response

**Figure 6:**
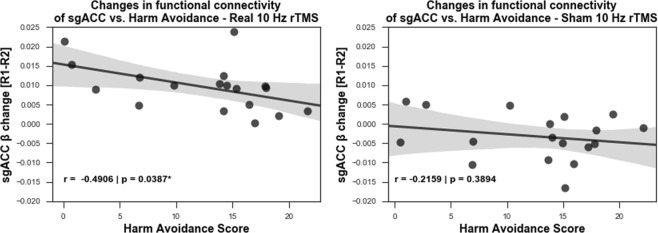
Harm avoidance score can predict rTMS induced reduction in functional connectivity of right sgACC (n = 18). A negative correlation between harm avoidance score and the reduction in the functional connectivity of the default mode network at the sgACC during R2 (27–32 min) from the R1 (10–15 min) rsfMRI windows, indicates that the lower the subject scores on the harm avoidance scale, the higher was the uncoupling resulting from rTMS.

The personality trait of HA has been suggested to predict therapeutic responses in MDD patients^28,29^. Therefore, considering the cumulative impact of multiple rTMS sessions for the treatment of depression^36^, we evaluated if HA scores would inform of the resulting effects of a single rTMS session in healthy volunteers. For that, we correlated HA scores from the TCI with the parameter estimates (beta weights) changes of functional connectivity at the sgACC (R2 – R1, Fig. 6). We identified a stronger negative correlation between HA and the rTMS effects (the decrease in functional connectivity strengths between the DMN and the right sgACC (Fig. 6)) only in the real condition (r = −0.4906, p = 0.0387) and not in the sham condition (r = −0.2159, p = 0.3894).

### Behavioural correlates of functional connectivity changes

**Figure 7:**
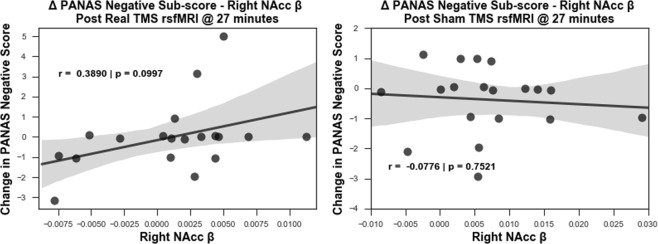
Subjects that perceived less negative affect after stimulation had lower functional connectivity in right NAcc (n = 19). A trend of positive correlation observed between the functional connectivity strength of the default mode network at the right NAcc and the changes in negative affect score on PANAS scale occurs in real rTMS (left) but not in sham rTMS (right) condition. The correlation shows a transient effect during R2 rsfMRI window (27–32 min) after rTMS: subjects who reported less negative emotions after stimulation showed less coupling between right NAcc and the default mode network.

Considering the role of the sgACC activity in sad mood^25,31,32^, we used the negative affect score from Positive and Negative Affect Schedule (PANAS), a psychometric scale that measures both positive and negative affect, to investigate behavioural outputs from real and sham rTMS. We explored the correlation between the changes in the negative affect scores (post stimulation – pre stimulation scores) with the R2 functional connectivity of the DMN to the right sgACC or to the right nucleus accumbens (NAcc). The latter was post hoc performed, since we observed robust decreases in the functional connectivity of right ventral striatum with DMN and this region is implicated in integrating emotional signals from limbic systems^37^. For that, we selected an independent coordinate for the right NAcc from the literature and extracted the beta weights. We report a trend of positive correlation (r = 0.3894, p = 0.0993) between the DMN-NAcc functional connectivity during R2 and changes in negative affect scores in the real condition, but not in the sham condition (Fig. 7).

## Discussion

Given the lack of studies exploring the mechanism of action of single session of FDA approved 10 Hz rTMS session in healthy volunteers, it is imperative to investigate mechanisms underlying such rTMS protocols, to bridge the existing knowledge gap in our basic understanding of HF-rTMS effects. The fact that about half of the patients receiving rTMS treatment for depression still do not clinically respond^38^ emphasizes the need to personalize rTMS stimulation. In an effort to improve rTMS target selection, which seems to strongly contribute to the observed variability in rTMS response^39–41^, we have proposed a novel and easy to implement, rsfMRI based rTMS target selection method for the left DLPFC. As a proof of concept we have shown in our study with healthy subjects, the target selection process yields reproducible results in the same individual generated from different datasets. By means of targeting left DLPFC sites with higher negative functional connectivity to sgACC, our results show that this target selection process holds potential for increasing rTMS therapeutic response in comparison to stimulating at MNI based anatomical left DLPFC coordinates. Upon employing this target selection method to investigate the mechanism of action of a full session of 10 Hz rTMS in healthy subjects, we show a reduced functional connectivity of the DMN with the sgACC after stimulation, and report for the first time, a reduced functional connectivity of this network with the vStr (both effects peaking at 27–32 minutes post HF-rTMS, namely R2). These results are particularly important as these regions are considered core structures implicated in the pathophysiology and treatment of depression^5–7,12,13,42^. Also, our study shows that it is possible to manipulate the functional connectivity of these deeper regions by stimulating at personalized cortical targets. Moreover, we have found that the lower the harm avoidance score, the stronger the HF-rTMS effects in the sgACC 27–32 minutes after stimulation. As the functional connectivity reduction of the DMN to the sgACC marked the effects driven by 10 Hz rTMS, we put forth the plausibility of using HA scores for anticipating sgACC responsiveness to HF-rTMS. Lastly, we have seen that the lower the functional connectivity of the DMN to the vStr, the less negative the subjects report their emotions. This correlation pattern was not identified in the sgACC. This hints at how the influence of 10 Hz rTMS on the DMN extends to behaviour, captured by the self-report of negative affect in healthy subjects.

Previous work has highlighted that stimulation sites displaying higher negative connectivity with the sgACC can achieve stronger antidepressant effects^22,23^, a characteristic that can be missed when selecting rTMS targets based on group averaging^43^. In this context and to achieve more cohesive results from stimulation in healthy volunteers, we developed a target selection process that incorporates individual functional connectivity characteristics from rsfMRI data. To establish the consistency of a method for personalized target selection in the DLPFC, one must first test whether target sites generated from different datasets of the same subject will coincide. Previous research on modelling of electric fields^33^ has shown that a figure-of-eight TMS coil covers a diameter of approximately 20 mm. Therefore, our method should yield left DLPFC targets that are within 20 mm of each other. Upon calculating the Euclidean distances between targets resulting from these two independent datasets, we have shown that the left DLPFC targets from independent datasets were on average 10.9 mm (6.74 mm SD) apart from each other. This indicates that even if the target might have slightly shifted on the day of stimulation, it largely remained within the 20 mm electric field. Thus, our approach maximizes the stimulation effects by capturing the interindividual variability of networks from a previous fMRI session and guiding the stimulation coil for each volunteer to the most promising site at the left DLPFC to manipulate the sgACC. We proved this important point by showing that individualized targets selected by our method (indDLPFC) have a higher overall negative correlation than the fixed left DLPFC target (fxdDLPFC) with the right sgACC (independent coordinates)^19^. Considering the anticorrelation of the sgACC and the stimulated DLFPC site quantifies the quality of HF-rTMS response^22,23^, we believe that our method can boost responses by guiding stimulation into these functionally relevant network nodes in comparison to anatomically guided stimulation.

**Figure 8:**
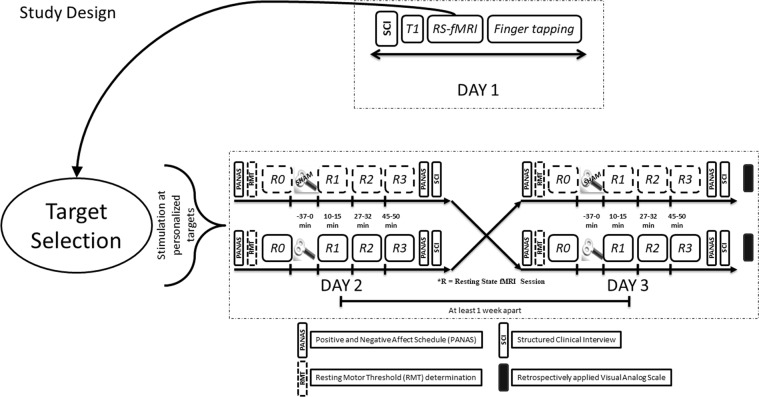
Study design – On day 1, after a Structured Clinical Interview (SCI) and obtaining informed consent, we acquired a T1 weighted structural image, rsfMRI and finger tapping task based fMRI. During rsfMRI, we instructed subjects to look at a fixation cross presented on a black background. The rsfMRI data was used for personalized target selection and the personalized target was then identified in T1 structural for stimulation using online neuronavigation. rsfMRI sessions were followed by an index finger tapping session (intermittent finger tapping when presented with a green or a red dot), data from which was used to determine the motor cortex location used for setting the resting motor threshold (RMT). At the beginning of experiment on day 2 and day 3, we asked each subject to complete the Positive and Negative Affect Schedule (PANAS). This was followed by resting motor threshold determination. We obtained a baseline rsfMRI scan (R0), and then delivered HF rTMS stimulation (either real or sham in a pseudo-randomized and counterbalanced way) to the subject at the pre-selected target, guided by online neuronavigation. Post HF rTMS three additional rsfMRI scans (R1, R2, and R3) were obtained over a course of 50 minutes: at 10 minutes, 27 minutes and 45 minutes to detect effects on brain resting state functional connectivity. The subjects again completed the PANAS immediately after leaving the MR scanner. This allowed us to document any short term changes in the self-rated emotional state. The subjects completed a SCI again at the end of experiment on both day 2 and day 3. To assess the effectiveness of sham blinding, we also retrospectively collected information about the perceived effects of stimulation on scalp sensation and mental state using a visual analog scale (VAS).

To understand the fundamental mechanism of 10 Hz rTMS protocol we delivered a complete (3000 pulses) session of personalized 10 Hz rTMS in healthy subjects. We found a robust decrease of functional connectivity after real HF-rTMS between the DMN and the sgACC as well as the vStr during R2. This decreased coupling persists during R3 in the sgACC, albeit less pronounced, but it returns to normal in the vStr during R3. Although with a similar study design as Tik and colleagues^19^, our results diverge from theirs. They reported an increase in the functional connectivity of the sgACC to a network consisting of the dorsal cingulate cortex, posterior dorsomedial prefrontal cortex, DLPFC, inferior parietal lobule, inferior frontal cortex and posterior temporal lobes. The difference in reported results potentially stems from the fact that we tracked HF-rTMS induced changes in the DMN for different and longer periods of time (during 10–15 (R1), 27–32 (R2), and 45–50 min (R3), compared to 15–21 and 31–37 min^19^) and investigated the DMN, which has been consistently described in the literature of depression^5–18,44^. We further argue that this discrepancy in results stems from the difference of networks that show an altered functional connectivity to sgACC. Tik *et al*.^19^ have reported a network different from the DMN having an increased functional connectivity to sgACC (see above for the regions included in their network RSN #17). Our study, on the other hand, has identified changes involving the DMN and compared it across four sequential rsfMRI sessions. Additional differences in TMS stimulation included reduced length of the FDA approved HF-rTMS protocol (1200 vs. 3000 pulses), reduced stimulation intensity (80% vs. 110% of Resting motor threshold - RMT), and different stimulation delivery (anatomical standard left DLPFC target vs. functional connectivity based personalized left DLPFC target), all of which could have contributed to a difference in results. Additional analysis of parameter estimates of left DLPFC stimulation sites in IC-DLFPC and sgACC in DMN reveals that the connectivity of left DLPFC decreases initially after stimulation from R0 to R1. This is followed by a decrease in functional connectivity of the sgACC to the DMN during a later time window (from R1 to R2). Hence, we report the differences in temporal dynamics of HF-rTMS induced changes observed at the site of stimulation (left DLPFC) and at more distant locations (sgACC).

Studies have observed higher sgACC functional connectivity in depression and its role in antidepressant response^6,11,45–55^. Further studies have shown abnormal sgACC connectivity with the DMN during depression^9,13,44^ and have reported decreased activity in sgACC in response to deep brain stimulation^52,56^. In this context, our results of decoupling the DMN and the sgACC after HF-rTMS in healthy subjects align well with such findings in patients with depression. However, since we have used one full session of HF-rTMS rather than 20 sessions, as is usually performed in clinical practice, this implication is limited in its interpretability. The NAcc, located at the vStr, is an integral component of the reward system and it was recently demonstrated^57^ that a disrupted reward circuit is associated with the pathophysiology of depression. Another study reported that the level of functional connectivity between the NAcc and left DLPFC target predicts the antidepressant response of HF-rTMS in depression^42^. Given the importance of the sgACC and the NAcc regions in the pathophysiology of depression, the changes reported in these regions after a full session in healthy volunteers hold significance for elucidating the underlying neural network mechanisms that result in antidepressant effects. In contrast to our report of decrease in functional connectivity of sgACC in the active stimulation condition only, Taylor *et al*.^18^ reported such a decrease across both sham and active HF-rTMS conditions. This discrepancy might stem from the fact that their results are from a patient population who were on a stable dose of medication, whereas ours are from healthy volunteers after a single session of personalised HF-rTMS.

In light of the changes of functional connectivity of sgACC reported here, and previous studies implicating sgACC and HA scores in healthy subjects^25–27^ we examined the correlations between HA and effects on functional connectivity of sgACC. The negative correlation between the harm avoidance scores and the changes in functional connectivity in the real stimulation condition, but not in sham indicates that harm avoidance score can potentially anticipate the extent of the right sgACC rTMS response in healthy subjects. This is an interesting finding in light of previous studies^28–30,58^, which reported that harm avoidance was a negative predictor of response to antidepressant treatment, i.e. patients with higher harm avoidance scores responded poorly to antidepressant treatment. Apart from this, Ward and colleagues^59^ also reported a weak link between poor antidepressant response and higher scores on neuroticism, which itself has been shown to positively correlate with harm avoidance scores^60^. Further studies^61,62^ also point towards a relation between high scores on neuroticism and low response to treatment of depression. Since our study is based on a cohort of healthy subjects, we believe harm avoidance of the TCI is a good proxy for neuroticism, given the positive correlation between the two personality measures. Hence, the correlation between HA and HF-rTMS effects in the sgACC reported here, warrants further clinical studies to investigate the possibility of using this personality dimension as a predictor for HF-rTMS driven antidepressant effects. Of note, previous HF-rTMS studies have reported persistence^63^ and self-directedness^64^ scores of the TCI as good predictors of antidepressant effects. Upon post-hoc analysis, we did not find such correlations between other TCI dimensions and HF-rTMS induced changes in the sgACC and this might be, since our sample consisted of healthy volunteers. Future studies in depression cohorts should address this issue.

As noted earlier, sgACC activity has been implicated in sad mood^31,32^ and rumination thoughts^13^ in healthy participants. We hence studied the relationship between the functional connectivity changes in sgACC and the reported negative emotional state using PANAS scale, which we administered before and after stimulation. However, we could not identify any relationship in sgACC with these measurements. Taking into consideration that the right vStr displayed robust reduction in functional connectivity during the same time window in which the sgACC decoupled from the DMN and that the NAcc is implicated in integrating emotional signals within the hubs of limbic system^37^, we investigated the relationship between this region and the reported negative emotional state of subjects. Although this relationship does not reach significance, there is a correlation trend only in the real HF-rTMS condition, which indicates that the lower the functional connectivity between the DMN and the right NAcc, the less negative subjects self-rated their emotions. The exact mechanism by which HF-rTMS might be influencing the negative percept of healthy subjects, however, requires further investigation.

We created a new method of personalized HF-rTMS target selection utilizing individual rsfMRI and used this approach to shed new light on the DMN changes resulting from of a single, complete dose of 10 Hz rTMS stimulation in healthy subjects. Since this translational approach has been initially implemented in healthy subjects only, it is obviously not possible to comment on the clinical efficacy of stimulating at such indDLPFC targets. Our study’s main goal was to delineate the underlying neural mechanisms of a full single dose of HF-rTMS protocol. Therefore, we chose sham over an alternative target selection process for direct comparison. This limits claims about how effective would the new personalization method be in the clinical practice, which will be tested in a new study. Multiple sessions of HF-rTMS might interact with brain networks differently than a single session, especially with the complexities and variations added upon by depression pathophysiology and treatment resistance. These questions remain to be further addressed in follow up studies. Another limiting aspect is that the sham condition was in fact an active sham condition, although with irrelevant current density, that did not result in functional connectivity differences. The use of a passive sham coil is unlikely to give different results from those presented here, but this should nevertheless be rigorously tested. Lastly, the correlation in self-rated emotional judgement was not statistically significant in the vStr and non-existent in the sgACC. We consider two possible explanations for that, one being that the sample size is limited in power, the other being that participants are healthy and only one session of HF-rTMS is insufficient to exhibit stronger effects. Nevertheless, new insights into the mechanisms associated with a full session of HF-rTMS can be gained from our results.

## Materials and Methods

### Participants

We recruited healthy male and female subjects between the ages of 18–65 with no current or prior psychiatric disorders, evaluated by structured clinical interviews (see below). Exclusion criteria were: current/history of neurological or psychiatric disorder, recreational drug use in the past month, current/history of substance abuse or dependence, contraindications to the MRI scanner (e.g. metal parts in the body) or TMS application (e.g. epilepsy), pregnancy, history of traumatic brain injury, unwillingness to consent or to be informed of incidental findings, current use of anticonvulsant drugs, or prior TMS or ECT application in the past 8 weeks. The Ethics Committee of the University of Medical Centre Göttingen has approved the study protocol. All experiments were performed in accordance with relevant guidelines and regulations^21,65^.

### Study design

We conducted a double-blind (subjects and interviewer), sham controlled, crossover study with healthy subjects. The experiments were conducted on 3 days with approximately one week between each appointment. Based on the resting state network pathophysiology of depression, we hypothesized that a single session of HF-rTMS would induce changes in the DMN^5^, which would be associated with self-reported behavioural changes in affect and with stable personality trait of harm avoidance.

#### Day 1

The subjects visited the lab on 3 occasions (day1, day2, and day3). On day 1 the subjects underwent a Structured Clinical Interview (SCI) consisting of Montgomery-Asberg Depression Rating Scale (MADRS), Beck Depression Inventory II (BDI II), Young Mania Rating Scale (YMRS), and Hamilton Depression Rating Scale (HAM-D). Apart from the SCI they also completed the Symptoms Checklist 90 (SCL 90), Positive and Negative Syndrome Scale (PANSS), Barratt Impulsiveness Scale (BIS), Temperament and Character Inventory (TCI), Life Orientation Test – Revised (LOT-R) and vocabulary intelligence test (MWT), and handedness questionnaire^66^. Upon being determined for inclusion/exclusion criteria, the subjects gave their written informed consent following which initial structural T1 and rsfMRI scans were recorded for our novel method of personalized target selection (see Fig. 8 for further details).

#### Day 2 and Day 3

Day 2 and day 3 were at least one week apart to allow wash out of any HF-rTMS stimulation effects. At the beginning of experiment on day 2 and day 3, we asked each subject to complete the PANAS. PANAS is composed of 20 items that measure positive and negative dimensions of affect. To evaluate negative affect we used the total raw score (1–5 points for each item) from the negative dimension items^67^.

After resting motor threshold (RMT) determination, we applied 10 Hz rTMS at personalized left DLPFC target, guided by an online neuronavigation system (Visor 1 software, ANT Neuro, Enschede, Netherlands) at 110% of RMT. We obtained a baseline rsfMRI scan (R0), and then delivered HF rTMS stimulation (either real or sham in a pseudo-randomized and counterbalanced way) to the subject at the pre-selected target, guided by online neuronavigation. In the event of extreme scalp discomfort, the stimulation was stopped and the subject was excluded from further experiments. Figure 8 pictorially details the study design.

### Target selection

For real and sham HF-rTMS on day 2 and day 3, we used the rsfMRI scan from day 1 to identify a personalized target in the left DLPFC for each subject, using a novel selection process as detailed in the Supplementary Information.

### rTMS Stimulation

We delivered 10 Hz rTMS using a MagVenture X100 with Mag-option and an MCF-B65 cooled butterfly coil. The stimulation parameters were as described in O’Reardon *et al*.^68^. To deliver sham stimulation, we rotated the coil by a full 180° along the handle axis of the coil.

To deliver sham stimulation, we rotated the coil by a full 180° along the handle axis of the coil such that the stimulation side of the coil faced away from the scalp and the distance between the stimulation side and the scalp was larger than 5 cm. We made measurements of the voltage induced on the “sham” side using a standard oscilloscope. The oscilloscope readings indicated that there was a very weak current strength produced by the sham side of the coil, and this negligible current did not elicit any motor responses, irrespective of how high the stimulator output was set to. Participants were blind to the control condition and simply received the information that we were testing two different rTMS stimulation coils.

### Imaging acquisition and analysis

We acquired the structural (T1- and T2-weighted scans with 1-mm isotropic resolution) and functional data with a 3T MR scanner (Magnetom TRIO, Siemens Healthcare, Erlangen, Germany) using a 32-channel head coil. The gradient-echo EPI sequence had the following parameters: TR of 2.5 seconds, TE of 33 ms, 60 slices with a multiband factor of 3, FOV of 210 mm × 210 mm, 2 × 2 × 2 mm, with 10% gap between slices and anterior to posterior phase encoding. The rsfMRI data was acquired with 125 volumes in approx. 5.5 minutes, whereas the finger tapping data was acquired with 103 volumes in approx. 4.5 minutes.

rsfMRI data analysis: Using SPM12 (http://www.fil.ion.ucl.ac.uk/spm/software/spm12/) and MATLAB (The MathWorks, Inc., Natick, MA, USA), we preprocessed the individual rsfMRI data using standard steps: slice time correction, motion correction, individual gradient echo field map unwarping, normalization, and regression of white matter, cerebrospinal fluid and motion nuisance parameters. We then temporally concatenated the data to perform group independent component analysis with FSL 5.0.7 software^69^. We identified the best fitting independent component (IC) that resembled the DMN. This IC was then back reconstructed in individual subjects’ normalized rsfMRI data, r-to-z transformed and compared across the groups (Real [R0, R1, R2, R3] versus Sham [R0, R1, R2, R3]).

Finger tapping fMRI data: We preprocessed the finger tapping data using slice time correction, motion correction, gradient echo field map-based distortion correction, co-registration to the anatomical scan and smoothing with an 8 mm FWHM kernel. The onset times and durations for green dot (finger tapping) and red dot (rest period) were extracted from log files generated by Presentation software (Neurobehavioral Systems, Inc.) to create a block design of the experiment. Estimates of neural activity were computed with a general linear model (GLM) for each subject individually using SPM12. First-level contrasts were calculated for the finger tapping and rest response blocks. By contrasting these blocks, we obtained the primary motor cortex areas activated by finger tapping.

Extraction of betas (functional connectivity strengths) from IC-ACC: To evaluate potential correlations between the left DLPFC and the sgACC in the IC-ACC, we compared the personalized targets with targets based on fixed MNI coordinates, as described in a recent study^19^. We used MarsBar^70^ to extract the parameter estimates (beta weights) utilizing an ROI of 2 mm radius around the personalized left DLPFC locations for real HF-rTMS stimulation, and around the standard MNI locations of the DLPFC and sgACC (as described in Tik *et al*.^19^), after transforming the coordinates into the individual anatomical spaces of our participants. To correlate functional connectivity strengths of sgACC and NAcc with the self-reported negative emotions, we utilized the same protocol as above to extract the parameter estimates of the right NAcc using a spherical ROI of 5 mm radius centred at [(10, 12, −8) based on Harvard-Oxford Sub-cortical Atlas of FSL] and of the right sgACC using a spherical ROI of 5 mm radius centred at [(8 40–6) based on coordinates reported in Tik *et al*.^19^]. The results presented are for instances in which the beta weights were successfully extracted using MarsBar.

### Statistical analysis

We used SPM12 to compare time windows of rsfMRI across real and sham conditions using a factorial design ANOVA, and only report results surviving a statistical threshold of whole brain p < 0.05 FWE correction for multiple testing. We used SPSS and Matlab to run two way t-tests to compare the scores from MADRS, HAM-D, YMRS, BDI II, PANAS, and VAS for real and sham stimulation sessions. Using MATLAB and R, we ran Pearson’s correlation tests between functional connectivity strengths of various brain regions and relevant behavioural scales.

## Supplementary information

Supplementary Information

## Data Availability

The datasets generated and analysed during the current study are not publicly available due to restrictions in the data sharing permissions obtained from study participants.
