# Supplementary material for: Personalized repetitive transcranial magnetic stimulation temporarily alters default mode network in healthy subjects
Source: Sci Rep. 2019 Apr 4;9:5631. doi: 10.1038/s41598-019-42067-3 (PMC6449366; doi:10.1038/s41598-019-42067-3)
Supplement: Supplementary file 1 — Supplementary Information [file 41598_2019_42067_MOESM1_ESM.docx]

**Title: Personalized repetitive transcranial magnetic stimulation temporarily alters default mode network in healthy subjects**

**Authors:** Aditya Singh^1^, Tracy Erwin-Grabner^1^, Grant Sutcliffe^1^, Andrea Antal^2^, Walter Paulus^2^, Roberto Goya-Maldonado^1,^*

**Affiliations:**

^1^Systems Neuroscience and Imaging in Psychiatry, Department of Psychiatry and Psychotherapy of the University Medical Center Göttingen.

^2^Department of Clinical Neurophysiology of the University Medical Center Göttingen.

*To whom correspondence should be addressed: Dr. Roberto Goya-Maldonado ([roberto.goya@med.uni-goettingen.de](mailto:roberto.goya@med.uni-goettingen.de))

**Supplementary Information**

**Supplementary figures:**

**Supplementary Figure 1**: **Scheme detailing the target selection process using a single subject as an example.**

*a. Individual subject rsfMRI preprocessing pipeline*. The resting state functional magnetic resonance imaging (rsfMRI) data from the individual subject is pre-processed. Using SPM12 (<http://www.fil.ion.ucl.ac.uk/spm/software/spm12/>[)](http://www.fil.ion.ucl.ac.uk/spm5)) and MATLAB (The MathWorks, Inc., Natick, MA, USA), we preprocessed the individual subject’s rsfMRI data using standard steps: slice time correction, motion correction, individual gradient echo field map unwarping (step 1), normalization (step 2.2), and regression of white matter, cerebrospinal fluid and motion nuisance parameters (step 3). We then temporally concatenated the data to perform group independent component analyses (ICA) as well as individual ICA using FSL 5.0.7 software^1^ (step 4). The number of independent components was restricted to 17 based on the literature^2^. We identified the best fitting independent components (IC) covering: 1) the left DLPFC (IC-DLPFC) and 2) the sgACC (IC-ACC). These components were identified based on visual inspection of the MELODIC results from FSL 5.0.7. All relevant ICs that covered the left DLPFC or the ACC in a complete or partial manner were identified. Thus, if more than one IC covering the left DLPFC or ACC was identified, all the selected ICs were carried forward to the next step of analysis.

*b. Individual subject target selection pipeline.* The ICs obtained prior were in standard MNI space. To reconstruct left DLPFC targets in individual brain spaces, we reran the preprocessing pipeline described above without normalizing the data, but instead co-registering it to the subject’s T1 image, and additionally smoothing the data with a FWHM Gaussian kernel size of 8 mm (step 1 + step 2.1). Next, we back reconstructed the IC-DLPFC and IC-ACC in the non-normalized rsfMRI data to obtain these components in the individual anatomical space. This was achieved by running a linear regression of the time course of the ICs on the non-normalized rsfMRI data using SPM12 (step 5).

As previously reported in the literature^3,4^, better clinical responses to HF-rTMS in patients with depression are associated with higher negative functional connectivity between the sgACC and stimulation target in the left DLPFC. To incorporate this feature into our personalized target selection for HF-rTMS, we overlaid individual negative correlation maps of the IC-ACC with the positive correlation maps of the IC-DLPFC (p<0.001) using Chris Rorden’s MRIcron (step 6). The negative correlation map of the IC-ACC was thresholded leniently (p<0.01) to allow the detection of viable overlaps. The positive correlation map of the IC-DLPFC was obtained by saving the contrast file of the corresponding IC-DLPFC with the contrast set to +1 in SPM 12. Similarly, we obtained the negative correlation map of IC-ACC by saving the contrast file with the contrast set to -1 in SPM 12. Next, we located a point within the overlap of these two maps and then identified the closest local maximum nodes (strongest node) of the IC-DLPFC to this point using SPM12 (step 7). As the last step, we confirmed that the local maxima still laid within the overlap and was designated as target of stimulation (step 8). In cases of subjects for whom multiple IC-DLPFC or IC-ACC were identified, we identified all nodes that lay within the overlap of the positive correlation map of IC-DLPFC and the negative correlation map of IC-ACC. In such cases, the strongest connectivity node within the IC-DLPFC (maximum t-value), that still lay within the overlap, was selected as the target for rTMS stimulation.

We thus incorporated two features that define an optimal target: the target is based on individual rsfMRI data^5^ rather than a group average and the target has a negative correlation to sgACC^3,4^.

Also, note that even though we ran a temporally concatenated ICA (tc-ICA) on a group of rsfMRI data that included the subject for which the target was sought, we used the ICs obtained from individual ICA results for target selection. We used the ICs from tc-ICA only in cases when ICs from individual ICA failed to yield any viable points, due to lack of an overlap. In the cases that we were unable to obtain an overlap using tc-ICA ICs, we repeated the day 1 rsfMRI measurement and applied target selection to the new data. For cases in which this was necessary (n = 3), we successfully identified targets with repeated rsfMRI measurement for all subjects.

**References**

1. Jenkinson, M., Beckmann, C. F., Behrens, T. E. J., Woolrich, M. W. & Smith, S. M. FSL. *Neuroimage* **62,** 782–790 (2012).

2. Yeo, B. T. T. *et al.* The organization of the human cerebral cortex estimated by intrinsic functional connectivity. *J. Neurophysiol.* **106,** 1125–1165 (2011).

3. Fox, M. D., Buckner, R. L., White, M. P., Greicius, M. D. & Pascual-Leone, A. Efficacy of transcranial magnetic stimulation targets for depression is related to intrinsic functional connectivity with the subgenual cingulate. *Biol. Psychiatry* **72,** 595–603 (2012).

4. Weigand, A. *et al.* Prospective Validation That Subgenual Connectivity Predicts Antidepressant Efficacy of Transcranial Magnetic Stimulation Sites. *Biol. Psychiatry* **84,** 28–37 (2018).

5. Fox, M. D., Liu, H. & Pascual-Leone, A. Identification of reproducible individualized targets for treatment of depression with TMS based on intrinsic connectivity. *Neuroimage* **66,** 151–160 (2013).

**
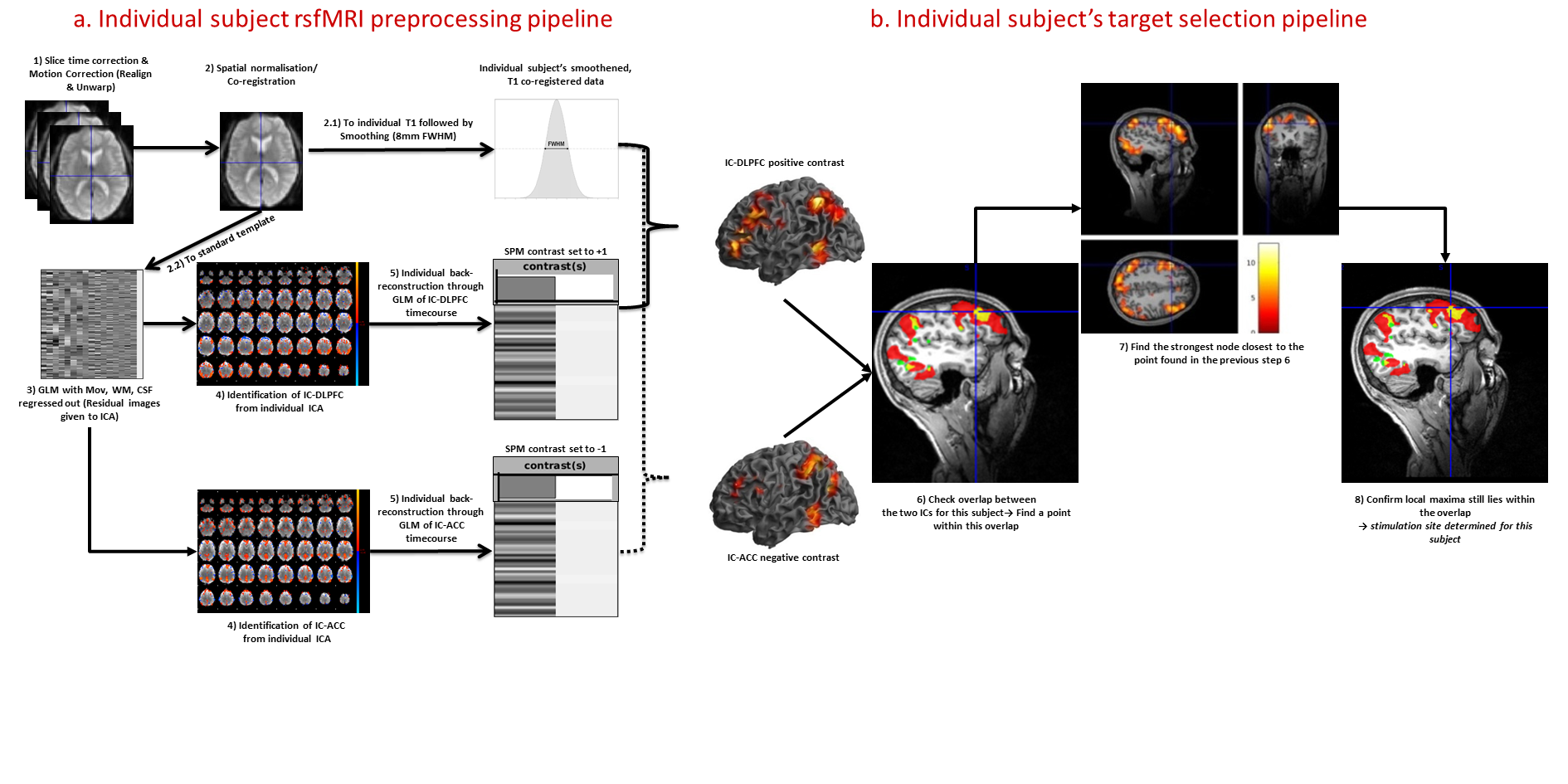
**
